# Is targeted biopsy necessary for Prostate Imaging Reporting and Data System 3 lesions?

**DOI:** 10.1590/1806-9282.20241958

**Published:** 2026-08-03

**Authors:** Diğdem Kuru Öz, Sezer Nil Yılmazer Zorlu, Nuray Haliloğlu, Ayşe Erden

**Affiliations:** 1 Ankara University, School of Medicine, Department of Radiology – Ankara, Turkey.; 2 Mamak State Hospital – Ankara, Turkey.

**Keywords:** Multiparametric magnetic resonance imaging, Prostate, Ultrasound, Biopsy

## Abstract

**OBJECTIVE:**

The aim of this study was to reveal the histopathological results of Prostate Imaging Reporting and Data System 3 lesions and to investigate whether targeted biopsy is essential in these lesions.

**METHODS:**

One hundred and twenty-seven patients with 176 lesions, being categorized as Prostate Imaging Reporting and Data System 3 according to Prostate Imaging Reporting and Data System version 2.1, who underwent transrectal ultrasound-magnetic resonance imaging fusion biopsy were included in the study.

**RESULTS:**

The mean age of the patients was 62.4 years. The median of prostate-specific antigen, prostate-specific antigen density, free prostate-specific antigen, and free/total prostate-specific antigen were 6.64 ng/mL, 0.10 ng/mL^2^, 1.24 ng/mL, and 0.18 ng/mL, respectively. In patient- and lesion-based analysis, the prevalence of adenocarcinoma was 12.7 and 9%, and clinically significant prostate cancer was 2.3 and 1.7%, respectively. The rate of prostate cancer detected by systematic biopsy alone was 7.8%, and 1.5% of these were clinically significant. There was no significant difference in prostate-specific antigen or prostate-specific antigen density between patients with histopathologically malignant and benign Prostate Imaging Reporting and Data System 3 lesions (p≥0.233). prostate-specific antigen density was higher in malignant patients, considering only 89 patients with index lesion P3 (p=0.046). Family history was significantly higher in malignant patients (p=0.028).

**CONCLUSION:**

Since clinically significant prostate cancer is very low in Prostate Imaging Reporting and Data System 3 lesions, clinical findings and risk factors should be considered in the biopsy decision in patients with index lesion Prostate Imaging Reporting and Data System 3. However, in patients with index lesions Prostate Imaging Reporting and Data System 4 and 5, targeting these Prostate Imaging Reporting and Data System 3 areas might be important with regard to the extent of the disease.

## INTRODUCTION

Patients with elevated prostate-specific antigen (PSA) levels, abnormal digital rectal examinations, or a family history of prostate cancer are often referred for multi-parametric magnetic resonance imaging (mpMRI). The Prostate Imaging Reporting and Data System (PI-RADS), now in its updated version (PI-RADSv2.1), aims to identify clinically significant prostate cancer (CSPC) while minimizing detection of low-risk disease and improving lesion localization before biopsy^1^. Traditional transrectal ultrasound (TRUS)-guided systematic biopsies often miss certain tumors, leading to a growing preference for transrectal ultrasound-magnetic resonance imaging (TRUS-MRI) fusion biopsies targeting mpMRI-detected lesions.

While PI-RADS scores 1 and 2 suggest a low cancer risk and typically do not require biopsy, scores 4 and 5 indicate a high risk, warranting fusion biopsy. PI-RADS 3 lesions, however, present a clinical challenge due to their intermediate risk, with variable detection rates (5–30%) for prostatic adenocarcinoma^2-4^. Some studies advocate follow-up mpMRI rather than immediate biopsy for these lesions^5^. This study aims to evaluate the histopathological outcomes of biopsied PI-RADS 3 lesions and assess the necessity of targeted biopsy in these cases.

## METHODS

The Institutional Ethics Committee approved this retrospective study protocol and waived informed consent (I5-365-21).

### Study subjects

We retrospectively analyzed 127 patients who underwent prostate-targeted biopsy in our department between January 2020 and April 2022. All patients had mpMRI within 3 months before biopsy due to clinical suspicion of prostate cancer (elevated PSA, abnormal digital rectal examination, or family history). Lesions were graded using PI-RADS v2.1 by two radiologists (D.K.Ö. and A.E.) in consensus, and patients with at least one PI-RADS 3 lesion were included.

Serum total and free PSA levels from the visit closest to the magnetic resonance imaging (MRI) were recorded. Total prostate volume (TPV) and intravesical prostatic protrusion (IPP) were measured, and PSA density was calculated. TPV was determined using the PI-RADS v2.1 ellipsoid formula^6^, while IPP was measured as the perpendicular distance of protruding prostatic tissue from the bladder base on midsagittal T2-weighted images^7^. PSA density was calculated as total PSA divided by TPV. Additional data, including prior prostate biopsies and family history of prostate cancer, were recorded. Clinically significant cancer was defined as a Gleason Score ≥7 (3+4) or a Prognostic Grade Group ≥2.

### Magnetic resonance imaging acquisition

All images were acquired on a 3-Tesla MR scanner (Verio; Siemens Medical Solutions, Erlangen, Germany) following a 6-h fast using a standard body matrix coil. The protocol included T2-weighted sagittal, coronal, and axial turbo spin-echo (TSE) images and axial diffusion-weighted imaging with b-values of 0, 1,000, and 2,000 mm²/s using a single-shot multi-slice echo-planar sequence. Gadolinium chelate (0.2 mL/kg) was administered intravenously at 2.5 mL/s, followed by a 15 mL saline flush, and dynamic images were acquired in the axial plane with a 3D fat-suppressed T1W volumetric interpolated breath-hold examination sequence.

### Targeted biopsy

All biopsies were conducted by two radiologists (N.H. and D.K.Ö.) using a General Electric Loqic S8 US machine (GE Healthcare, Milwaukee, WI, USA). TRUS-MRI fusion biopsy was performed for lesions with a PI-RADS score ≥3, with at least two cores taken from each lesion. Additionally, a 12-core systematic biopsy was obtained from the medial and lateral sides of the apex, mid, and base regions of both prostate lobes.

### Statistical analysis

Statistical analysis was performed using SPSS v11.5 (SPSS Inc.). Quantitative variables were summarized as mean±standard deviation or median (min–max), while qualitative variables were presented as frequency (percentage). The t-test and Mann-Whitney U test were used for quantitative variables, and the chi-square test for qualitative variables. A p<0.05 was considered statistically significant.

## RESULTS

There were a total of 176 PI-RADS 3 lesions in 127 patients with a mean age of 62.4±7.2 years (age range 45–84 years). The median of PSA, PSA density, free PSA, and free/total PSA values were 6.64 ng/mL, 0.10 ng/mL^2^, 1.24 ng/mL, and 0.18 ng/mL, respectively. Forty-three patients (33.8%) had a history of at least one previous systematic prostate biopsy. Fifteen patients (11.8%) had a family history of prostate cancer.

Demographic data and clinical characteristics of patients are listed in Table 1.

**Table 1:** Demographic data and clinical characteristics of patients.

Age (mean±SD)	62.4±7.2 years	
	Median	Minimum–maximum
TPV (mL)	68	11–223
IPP (mm)	3.2	0–30
PSA (ng/mL)	6.64	0.86–28.2
PSA density (ng/mL^2^)	0.10	0.03–1.77
fPSA (ng/mL)	1.24	0.37–6.5
f/t PSA	0.18	0.06–0.46
	Frequency (n, %)	
Previous systematic prostate biopsy	43 (34.1)	
Family history	15 (27.3)	

TPV: total prostate volume; IPP: intravesical prostatic protrusion; PSA: prostate-specific antigen; fPSA: free prostate-specific antigen; f/tPSA: free/total prostate-specific antigen; SD: standard deviation.

PI-RADS 3 lesions were most commonly located on the right lobe (46.5%), at the midgland level (43.9%), and in the peripheral zone (71.9%). The mean size of the lesions was 10 mm (5–20 mm).

### Patient- and lesion-based analysis

In patient-based analysis, adenocarcinoma was detected in 16 (12.5%) of the 127 patients, and premalignant lesions characterized by high-grade prostatic intraepithelial neoplasia were detected in 3 (2.3%) patients. The index lesion was PI-RADS 3 in 89 (70.0%) patients, PI-RADS 4 in 31 (24.4%) patients, and PI-RADS 5 in 7 (5.5%) patients. Prostate cancer was detected in 6 of 89 patients with index lesion PI-RADS 3, and the histopathology result of the remaining 83 patients was benign.

There was no statistically significant difference between the patients with benign and malignant PI-RADS 3 lesions in terms of PSA, PSA density, freePSA, and free/total PSA levels (p>0.233) but there was a significant difference in terms of family history (p=0.028) (Table 2). In addition, when 89 patients with index lesion PI-RADS 3 were evaluated, a significant difference was found in terms of PSA density between patients with benign and malignant histopathology results (p=0.046).

**Table 2:** Comparison of clinical parameters of patients with histopathologically benign and malignant Prostate Imaging Reporting and Data System 3 lesions.

Clinic parameter Median (min–max)	Benign	Malignant	p
PSA	6.5 0.86–28.2	8.84 4.02–14.11	0.319
PSA density	0.1 0.03–1.77	0.11 0.06–0.43	0.451
fPSA	1.14 0.37–6.5	1.38 0.44–2.44	0.703
f/tPSA	0.19 0.06–0.46	0.16 0.09–0.26	0.233
Family history*(n, %)	10 (9)	5 (31.2)	0.028**

* Frequency.

** Statistically significant.

PSA: prostate-specific antigen, fPSA: free prostate-specific antigen, f/tPSA: free/total prostate-specific antigen.

Even though the PI-RADS 3 lesion was benign, in 10 of the 127 patients (7.8%), adenocarcinoma was detected only by systematic biopsy, with 2 (1.5%) of these being CSPC (Gleason Score [GS] 7, Prognostic Grade Group [PGG] 3 and GS 7, PGG 2).

A total of 176 PI-RADS 3 lesions were detected in 127 patients.

**Figure 1:**
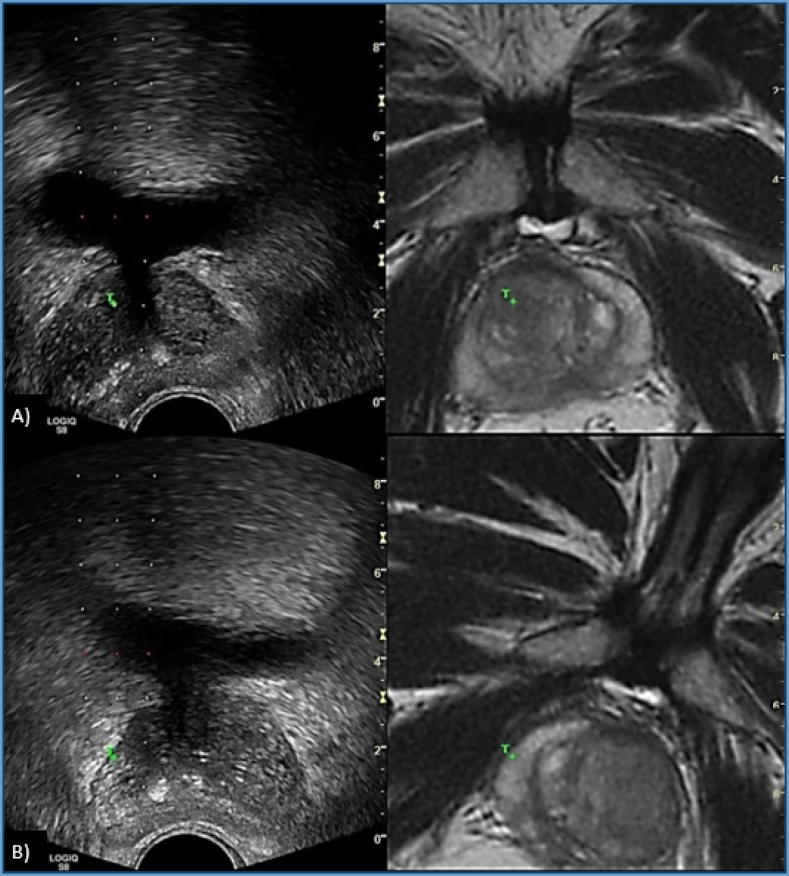
Magnetic resonance imaging-transrectal ultrasound fusion biopsy images of a 67-year-old man with a prostate-specific antigen value of 14.11 ng/ml, index lesion in category Prostate Imaging Reporting and Data System 5 located on the right transition zone at the base (A) and the lesion evaluated in category Prostate Imaging Reporting and Data System 3 on the right peripheral zone posterolateral at the apex (B). Both Prostate Imaging Reporting and Data System 5 index lesion and Prostate Imaging Reporting and Data System 3 lesion were histopathologically Gleason score 7 (4+3), Prognostic Grade Group 3. T: target; GS: Gleason score; PGG: Prognostic Group Grade.

**Figure 2:**
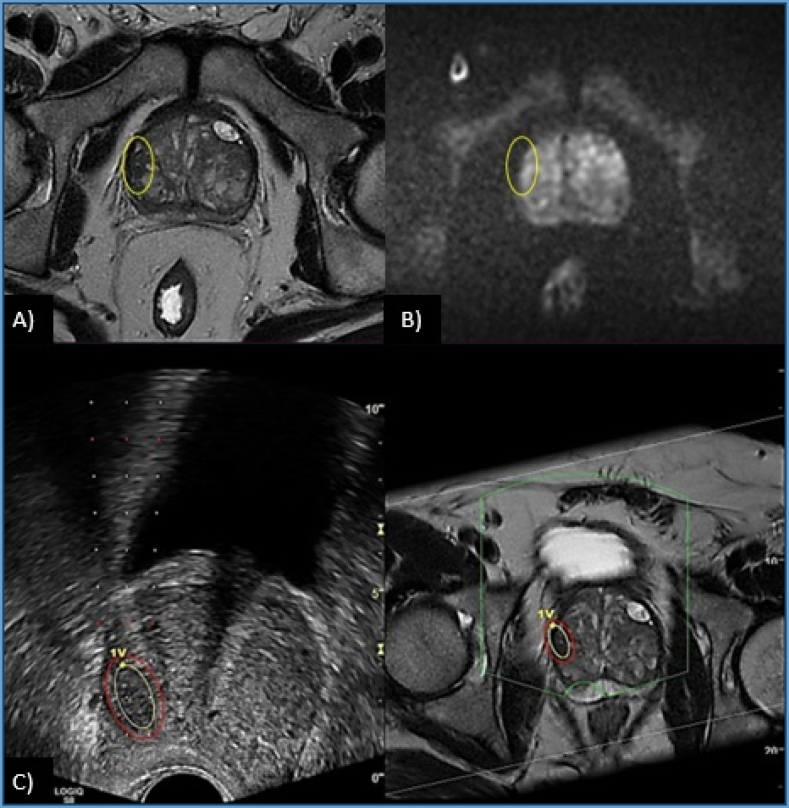
A 65-year-old man with prostate-specific antigen 9.71, on T2 AG (A) a slightly irregularly circumscribed hypointense area in the right transition zone at the midgland and on diffusion-weighted image with b=2000 s/mm2 (B), a mild hyperintense signal in this area showing diffusion restriction (apparent diffusion coefficient map not shown). MR-transrectal ultrasound fusion biopsy image (C) of the index lesion evaluated as Prostate Imaging Reporting and Data System 3. The index Prostate Imaging Reporting and Data System 3 lesion was Gleason score 4+3 Prognostic Grade Group histopathologically.

In lesion-based-analysis; The histopathological examination revealed adenocarcinoma in 16 (9%) of the 176 lesions; with a GS 6 (3+3), PGG 1 in 13 (7.4%) lesions, GS 7 (4+3), PGG 3 in 2 (1.1%) lesions, and GS 7 (3+4), PGG 2 in 1 (0.5%) lesion. There were three lesions with CSPC (patient- and lesion-based analysis, 2.3 and 1.7%, respectively). The accompanying index lesion was PI-RADS 5 in one of the three patients with CSPC with a PI-RADS 3 lesion (Figure 1), while the index lesion was PI-RADS 3 in the other two (Figure 2). The PI-RADS category of the accompanying index lesion in malignant PI-RADS 3 lesions and the pathology results are given in Table 3. The index lesion was PI-RADS 4 in 9 of 13 patients with clinically insignificant adenocarcinoma, and the index lesion was P3 in 4 patients. In 6 of 9 patients with an index lesion, PI-RADS 4 had adenocarcinoma with GS 6 (3+3), PGG 1.

**Table 3:** The Prostate Imaging Reporting and Data System category of the accompanying index lesion in malignant Prostate Imaging Reporting and Data System 3 lesions and the pathology results.

Cases	Index lesion PI-RADS category	Index lesion pathology	PI-RADS 3 lesion pathology
1	PI-RADS 5	GS 7 (4+3), PGG 3	GS 7 (4+3), PGG 3
2	PI-RADS 3	GS 7 (4+3), PGG 3	
3	PI-RADS 3	GS 7 (3+4), PGG 2	
4	PI-RADS 4	GS 6 (3+3), PGG 1	GS 6 (3+3), PGG 1
5	PI-RADS 4	Severe prostatitis	GS 6 (3+3), PGG 1
6	PI-RADS 4	GS 6 (3+3), PGG 1	GS 6 (3+3), PGG 1
7	PI-RADS 4	GS 6 (3+3), PGG 1	GS 6 (3+3), PGG 1
8	PI-RADS 4	GS 6 (3+3), PGG 1	GS 6 (3+3), PGG 1
9	PI-RADS 4	GS 6 (3+3), PGG 1	GS 6 (3+3), PGG 1
10	PI-RADS 4	GS 6 (3+3), PGG 1	GS 6 (3+3), PGG 1
11	PI-RADS 4	Prostatitis, atrophy	GS 6 (3+3), PGG 1
12	PI-RADS 4	Severe prostatitis	GS 6 (3+3), PGG 1
13	PI-RADS 3	GS 6 (3+3), PGG 1	
14	PI-RADS 3	GS 6 (3+3), PGG 1	
15	PI-RADS 3	GS 6 (3+3), PGG 1	
16	PI-RADS 3	GS 6 (3+3), PGG 1	

GS: Gleason score, PGG: Prognostic Group Grade; PI-RADS: Prostate Imaging Reporting and Data System.

In 160 (90.9%) of the 176 PI-RADS 3 lesions, histopathological examination demonstrated benign findings, including benign prostate tissue, prostatitis, and atrophy.

## DISCUSSION

Prostate cancer screening primarily relies on serum PSA testing, but its sensitivity and specificity are low due to confounding factors like benign prostatic hyperplasia and prostatitis. While mpMRI is not a standard screening tool, it is often reserved for high-risk patients or those with negative TRUS biopsy but rising PSA levels^8^. Due to the high negative predictive value of MRI, targeted biopsy is not recommended for PI-RADS 1-2 lesions, while PI-RADS 4–5 lesions warrant TRUS-MR fusion biopsy, with or without systematic biopsy^9-13^. However, the management of PI-RADS 3 lesions remains uncertain.

Studies have shown variability in CSPC prevalence for PI-RADS 3 lesions due to differences in population disease prevalence, MRI quality, reader expertise, biopsy methods, and histological factors^11^. A meta-analysis of 13 studies using PI-RADS v2 reported a 12% CSPC prevalence in biopsy-naive patients^12^. Other studies found rates ranging from 20 to 31% depending on biopsy techniques and study designs^11,14,15^. In our study, both the prevalence of prostate cancer and CSPC in PI-RADS 3 lesions were lower than the literature in both patient and lesion-based analysis. Several factors may explain this lower CSPC detection rate in our cohort. First, our study utilized PI-RADSv2.1, which includes updated criteria particularly for transitional zone lesions and allows for upgrading lesions based on diffusion-weighted imaging. This version may result in more conservative classification of lesions as PI-RADS 3 compared to the earlier PI-RADS v2 used in most aforementioned studies published before 2019. Second, we observed that increasing radiologist experience over time contributed to more precise lesion characterization, resulting in fewer PI-RADS 3 reports in our clinical practice. We assume that differences in reader experience levels might be another underlying cause for inter-study discrepancies. Third, our cohort included a significant proportion of patients with low PSA density, and this factor, combined with varying biopsy-naive status, may have influenced the overall cancer prevalence in our population.

PI-RADS guidelines provide no clear management strategy for category 3 lesions. Clinical factors, such as PSA density, serum biomarkers, and risk calculators, can stratify CSPC risk^16^. Studies suggest a PSA density threshold of 0.10 ng/mL² may guide biopsy decisions^17-19^. In our cohort, PSA density differences were significant when analyzing only patients with PI-RADS 3 index lesions, highlighting its potential predictive value.

Family history is a significant prostate cancer risk factor^20-22^. Consistent with prior studies, our findings suggest family history may justify targeted biopsy for PI-RADS 3 lesions. PI-RADS guidelines aim to balance reducing low-risk cancer diagnoses with enhancing CSPC detection.

In our study, the prevalence of clinically insignificant prostate cancer was 7.8%, lower than the reported range of 17–37% in combined biopsy approaches^23^.

The clinical implications of our findings suggest that PSA density serves as a valuable stratification tool for biopsy decision-making in PI-RADS 3 lesions, particularly when considering patients with index PI-RADS 3 lesions. Additionally, integrating family history should be emphasized when evaluating biopsy necessity in this subgroup. Furthermore, when targeting PI-RADS 4 or 5 lesions, concurrent sampling of PI-RADS 3 areas may improve disease staging and influence treatment planning decisions.

Our study has several limitations and strengths that should be acknowledged. Limitations include the retrospective design, relatively small sample size, and lack of multivariate analysis to assess clinical factors influencing biopsy decisions. Additionally, we did not evaluate interobserver agreement for PI-RADS scoring, which could impact reproducibility. However, strengths of our study include the use of combined targeted and systematic biopsy protocols, consensus PI-RADS scoring by experienced radiologists, and comprehensive data collection regarding PSA parameters and family history, which enhance the clinical relevance of our findings.

## CONCLUSION

Given the low prevalence of CSPC in PI-RADS 3 lesions observed in our study, a selective approach to targeted biopsy is warranted for these equivocal lesions. The biopsy decision should integrate multiple clinical factors, including PSA density, family history of prostate cancer, and digital rectal examination findings, rather than relying solely on imaging characteristics. Centers should consider their own institutional malignancy rates and reader experience when developing protocols for PI-RADS 3 lesion management. However, when patients present with concurrent PI-RADS 4 or 5 index lesions requiring targeted biopsy, sampling of accompanying PI-RADS 3 areas remains important for comprehensive disease staging and treatment planning. This stratified approach may help optimize the balance between cancer detection and overdiagnosis while maintaining clinical effectiveness.

## Data Availability

The datasets generated and/or analyzed during the current study are available from the corresponding author upon reasonable request.

## References

[1] Padhani AR, Barentsz J, Villeirs G, Rosenkrantz AB, Margolis DJ, Turkbey B (2019). PI-RADS Steering Committee: The PI-RADS multiparametric MRI and MRI-directed biopsy pathway. Radiology.

[2] Liddell H, Jyoti R, Haxhimolla HZ (2015). mp-MRI prostate characterised PIRADS 3 lesions are associated with a low risk of clinically significant prostate cancer - a retrospective review of 92 biopsied PIRADS 3 lesions. Curr Urol.

[3] Verma S, Choyke PL, Eberhardt SC, Oto A, Tempany CM, Turkbey B (2017). The current state of MR ımaging-targeted biopsy techniques for detection of prostate cancer. Radiology.

[4] Kızılay F, Çelik S, Sözen S, Özveren B, Eskiçorapçı S, Özgen M (2020). Correlation of Prostate-Imaging Reporting and Data Scoring System scoring on multiparametric prostate magnetic resonance imaging with histopathological factors in radical prostatectomy material in Turkish prostate cancer patients: a multicenter study of the Urooncology Association. Prostate Int.

[5] Steinkohl F, Gruber L, Bektic J, Nagele U, Aigner F, Herrmann TRW (2018). Retrospective analysis of the development of PIRADS 3 lesions over time: when is a follow-up MRI reasonable?. World J Urol.

[6] Mythreyi C, Lauren H, Dipleen K (2019). Prostate Imaging-Reporting and Data System 2019 Version 2.1.

[7] Nose H, Foo KT, Lim KB, Yokoyama T, Ozawa H, Kumon H (2005). Accuracy of two noninvasive methods of diagnosing bladder outlet obstruction using ultrasonography: intravesical prostatic protrusion and velocity-flow video urodynamics. Urology.

[8] Lo GC, Margolis DJA (2020). Prostate MRI with PI-RADS v2.1: initial detection and active surveillance. Abdom Radiol (NY).

[9] Moldovan PC, Broeck T, Sylvester R, Marconi L, Bellmunt J, Bergh RCN (2017). What Is the negative predictive value of multiparametric magnetic resonance ımaging in excluding prostate cancer at biopsy? A systematic review and meta-analysis from the European Association of Urology Prostate Cancer Guidelines Panel. Eur Urol.

[10] Visschere PJ, Naesens L, Libbrecht L, Praet C, Lumen N, Fonteyne V (2016). What kind of prostate cancers do we miss on multiparametric magnetic resonance imaging?. Eur Radiol.

[11] Leest M, Cornel E, Israël B, Hendriks R, Padhani AR, Hoogenboom M (2019). Head-to-head comparison of transrectal ultrasound-guided prostate biopsy versus multiparametric prostate resonance ımaging with subsequent magnetic resonance-guided biopsy in biopsy-naïve men with elevated prostate-specific antigen: a large prospective multicenter clinical study. Eur Urol.

[12] Barkovich EJ, Shankar PR, Westphalen AC (2019). A Systematic review of the Existing Prostate Imaging Reporting and Data System Version 2 (PI-RADSv2) literature and subset meta-analysis of PI-RADSv2 categories stratified by gleason scores. AJR Am J Roentgenol.

[13] Panebianco V, Barchetti G, Simone G, Monte M, Ciardi A, Grompone MD (2018). Negative multiparametric magnetic resonance ımaging for prostate cancer: what's next?. Eur Urol.

[14] Schoots IG (2018). MRI in early prostate cancer detection: how to manage indeterminate or equivocal PI-RADS 3 lesions?. Transl Androl Urol.

[15] Rouvière O, Puech P, Renard-Penna R, Claudon M, Roy C, Mège-Lechevallier F (2019). Use of prostate systematic and targeted biopsy on the basis of multiparametric MRI in biopsy-naive patients (MRI-FIRST): a prospective, multicentre, paired diagnostic study. Lancet Oncol.

[16] Veneziano S, Pavlica P, Compagnone G, Martorana G (2005). Usefulness of the (F/T)/PSA density ratio to detect prostate cancer. Urol Int.

[17] Hansen NL, Koo BC, Warren AY, Kastner C, Barrett T (2017). Sub-differentiating equivocal PI-RADS-3 lesions in multiparametric magnetic resonance imaging of the prostate to improve cancer detection. Eur J Radiol.

[18] Girometti R, Giannarini G, Panebianco V, Maresca S, Cereser L, Martino M (2022). Comparison of different thresholds of PSA density for risk stratification of PI-RADSv2.1 categories on prostate MRI. Br J Radiol.

[19] Görtz M, Radtke JP, Hatiboglu G, Schütz V, Tosev G, Güttlein M (2021). The value of prostate-specific antigen density for Prostate Imaging-Reporting and Data System 3 lesions on multiparametric magnetic resonance ımaging: a strategy to avoid unnecessary prostate biopsies. Eur Urol Focus.

[20] Steinberg GD, Carter BS, Beaty TH, Childs B, Walsh PC (1990). Family history and the risk of prostate cancer. Prostate.

[21] Nair-Shalliker V, Bang A, Egger S, Yu XQ, Chiam K, Steinberg J (2022). Family history, obesity, urological factors and diabetic medications and their associations with risk of prostate cancer diagnosis in a large prospective study. Br J Cancer.

[22] Madersbacher S, Alcaraz A, Emberton M, Hammerer P, Ponholzer A, Schröder FH (2011). The influence of family history on prostate cancer risk: implications for clinical management. BJU Int.

[23] Stabile A, Giganti F, Emberton M, Moore CM (2018). MRI in prostate cancer diagnosis: do we need to add standard sampling? A review of the last 5 years. Prostate Cancer Prostatic Dis.

